# Sonographic Evaluation of an Inguinal Bubo from *Bartonella henselae*: A Case Report

**DOI:** 10.5811/cpcem.47934

**Published:** 2025-11-16

**Authors:** Julian Campillo Luna, Robert W. Shaffer

**Affiliations:** University of Michigan, Emergency Department, Ann Arbor, Michigan

**Keywords:** *ultrasound*, *emergency medicine*, Bartonella, *bubo*, *case report*

## Abstract

**Introduction:**

Emergency physicians can use point-of-care ultrasound (POCUS) to identify lymph nodes in certain clinical scenarios, and advanced users can determine significant information (such as concerns for malignancy or differentiating them from abscesses for incision and drainage) based on a large volume of literature and images associated with those pathologies.^1^ However, current literature does not contain a similar volume of images and cases of suppurative lymph nodes, or buboes, limiting the ability to make the diagnosis sonographically at the bedside.

**Case Report:**

We report on a man who presented to the emergency department (ED) with a worsening inguinal mass that changed size with positioning, as well as a 20-pound weight loss occurring over the course of a month. Point-of-care ultrasound of the mass was concerning for a necrotic suppurative lymph node, which was further evaluated with cross-sectional imaging. The patient was admitted for a biopsy to rule out malignancy. He was discharged with serologies for *Bartonella henselae* pending, which later returned positive. The patient was then switched to azithromycin with significant improvement of his symptoms.

**Conclusion:**

As POCUS becomes the modality of choice for rapid assessment of soft tissue masses in the ED, familiarity with less common variants of soft tissue infections such as buboes can help with medical decision-making, risk stratification, and further workup. This sonographic description of a bubo caused by a common zoonotic infection will enable clinicians to familiarize themselves with their appearance.

## INTRODUCTION

Buboes are suppurative and necrotic lymph nodes with significant associated tenderness. First described in the sixth century during the Justinian plague,^2^ they were frequently encountered as the manifestations of *Yersinia pestis* infections, a disease often referred to as “bubonic plague.” However, *Y. pestis* is not the only causative agent of buboes; they have been described in a range of diseases from venereal infections such as *Chlamydia trachomatis*, *Haemophilus ducreyi*, and syphilis,^3^ as well as mycobacterial infections (both classic tuberculosis and atypical mycobacteria),^4^ zoonotic diseases such as bartonellosis (cat scratch disease), filariasis, and tularemia,^5^ and non-infectious diseases such as cancer.^6^

Buboes often represent a natural progression of localized infection of bacterial pathogens into the lymphatic system, creating a localized response that then produces purulence secondary to neutrophil migration. The diagnosis of a bubo is made clinically, although imaging and histological examination can greatly aid in the diagnosis, as the differential for these lesions is broad. Their location, commonly near large neurovascular bundles (axillary, inguinal, and cervical), means clinicians must also rule out malignancy, vascular abnormalities such as pseudoaneurysms, and uncomplicated abscesses. Point-of-care ultrasonography (POCUS) in the hands of an apt clinician lends itself to rapid evaluation of most or all these etiologies.

## CASE REPORT

A 30-year-old male with no significant past medical history presented to the emergency department (ED) with a chief complaint of a soft-tissue mass on his abdomen that changed size depending on his positioning, getting bigger when he stood and decreasing in size when he would lie down. This mass was in the left groin area and had been growing for a month. Over the preceding week the patient had also noticed a small amount of erythema overlying the mass, and it had become painful at rest and tender to touch. He also reported approximately 20 pounds of weight loss over the prior month and a half, which made him worry that he had cancer.

He initially presented to his primary care physician (PCP), who evaluated him with sexually transmitted infection serologies, which were negative, as well as an outpatient ultrasound that demonstrated enlarged, hypoechoic, and hyperemic left-groin lymph nodes with a 3.7-cm complex fluid collection, which was concerning for an abscess. He did not undergo incision and draining. He then presented to his local ED where he was further evaluated with computed tomography (CT) of his abdomen and pelvis with intravenous contrast and found to have a 4.7-cm nonspecific, soft-tissue nodule in the left inguinal region, likely an infected or inflammatory lymph node. He was then discharged with seven days of cephalexin.

He presented to our ED approximately six days into his cephalexin course, with concerns that the mass kept growing despite the antibiotics. On exam he presented with an enlarged inguinal mass with mild erythema (Image).

The patient was evaluated with a basic laboratory panel including a complete blood count, a basic metabolic panel, a urinalysis, lactate dehydrogenase due to concerns for malignancy, C-reactive protein, and a set of blood cultures, all of which were within normal range. A radiology-based ultrasound was ordered, which re-demonstrated a complex fluid collection below the skin concerning for abscess. We also ordered computed tomography (CT) of the abdomen and pelvis to compare to the prior image to determine whether further growth had occurred, and to ensure that this was not a small hernia given its changes with position. The CT confirmed interval increase in soft tissue hypodense abnormality from 1.7 cm to 3.9 cm. The read was concerning for either a septation or vascularity, which led us to obtain POCUS. Using a high-frequency linear probe, the L20-5s (Mindray Medical International Ltd, Shenzhen, China), we were able to visualize the lesion in greater detail.

We saw a complex fluid collection, with a thin strip of echogenic material nearly bisecting it longitudinally. A small defect in that echogenic material had created a pathway for the slightly hypoechoic fluid inside to flow from deep to superficial, which we were able to demonstrate with gentle compression of the skin overlying the area of maximum fluctuance (Video). This was interpreted at bedside to be purulent fluid moving across the space.

These findings led us to believe it was not a simple abscess and to continue our workup prior to an incision and drainage. We became concerned that POCUS had instead revealed a bubo. Further questioning of the patient revealed that he had recently acquired a kitten, which would often scratch at his legs. No abrasions were noted to the legs of the patient to suggest a bacterial, soft tissue infection as the cause of lymph node reactivity, but this information increased our clinical suspicion for cat scratch disease, and *B. henselae* antibodies were sent at that time.

*CPC-EM Capsule*

What do we already know about this clinical entity?

*Buboes can present in multiple different illnesses, among them bartonellosis. Ultrasound can aid in rapidly evaluating soft-tissue masses.*

What makes this presentation of disease reportable?

*To our knowledge, this is the first description of the sonographic appearance of a bubo from Bartonella henselae.*

What is the major learning point?

*This report identifies the sonographic features of an inguinal bubo and how it differs from soft-tissue abscesses and masses.*

How might this improve emergency medicine practice?

*By empowering physicians to recognize the morphological appearance of these buboes, painful and unnecessary incision and drainage procedures can be avoided.*

The patient was admitted to our short-stay medical unit where we completed the workup, specifically to rule out malignancy, while waiting for the *Bartonella* titers. He received an interventional radiology-guided needle biopsy of the mass, which was unrevealing. At that point, the patient was discharged with a seven-day prescription for trimethoprim-sulfamethoxazole (TMP-SMX) and instructed to follow up with the infectious diseases clinic as an outpatient. The day after discharge the patient’s titers returned positive for *B, henselae* immunoglobulin G at a 1:4096 titer (reference range: less than 1:128) suggesting recent/active infection. His tuberculosis serology was negative, and his needle biopsy showed lymph node tissue with no neoplastic changes. Fluid cultures were positive for *Enterococcus faecalis* in small quantities, favored to be a contaminant by the infectious diseases team following the case. The patient was informed of these findings; however, he missed his initial appointment with infectious diseases.

The patient called his PCP after the TMP-SMX course had been completed, with failure to improve. After a phone conversation with the infectious diseases team, the patient was switched to azithromycin (500 mg on day one and 250 mg daily for four additional days) per US Centers for Disease Control and Prevention recommendations. Follow-up a couple days later with his PCP showed that the patient’s pain was decreasing significantly. He presented to the infectious diseases clinic a week later with complete resolution of the symptoms. He has not since followed up or interacted with our medical system.

## DISCUSSION

This case is a reminder that the bedside assessment for abscess and soft tissue infection using POCUS can go further than determining whether a collection of fluid exists or whether vascular features are present. As ultrasound equipment becomes more advanced and physicians become more adept using it, POCUS can serve as a useful tool for further narrowing down a differential for these clinical cases. In this case ultrasonography was critical in accelerating care and eventually in accurately diagnosing the patient. The differential for bubo-producing illnesses includes significant diseases of public health note such as syphilis, chlamydia, and bubonic plague caused by *Y. pestis*. It can also be caused by lymphoma, as well as *B. henselae.*

In this case, the patient experienced changes in the size and protuberance of this mass with positioning and bearing down, which combined with the location and tenderness of the mass, made us significantly concerned for a hernia. Ultrasound demonstrated that the purulent material was traveling through a defect in the internal architecture of the lymph node, causing more pus to collect on the outside section as the patient’s intra-abdominal pressure increased. This is clearly seen in the video, during which graded compression achieved this same result in the opposite direction. Interestingly, the literature contains other cases of buboes masquerading as hernias due to this same effect, including one in which the organism was identified as *Mycobacterium avium intracellulare.*^4^

## CONCLUSION

Ultrasonographic assessment of soft tissue masses and collections can go further than assessing for vascular structures in or over the planned incision-and-drainage site and help to significantly narrow the differential for an infectious process. This case is also a good reminder of the need to keep a broad differential for soft tissue masses. Emergency physicians should maintain buboes and their associated causal agents high in the differential for any abscess that fails to respond to traditional antibiotic therapy and have a low threshold for pursuing inpatient workups given the high-risk nature of these illnesses to both patient and public health.

## Figures and Tables

**Image:**
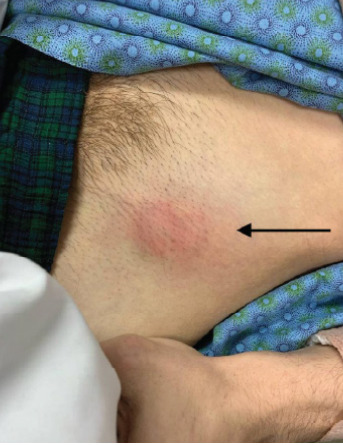
Photograph of patient’s left hip/inguinal region, with his feet facing the left of the image while supine. A 3-cm area of erythema and fluctuance was appreciated, corresponding to the patient’s source of pain (arrow).

**Video:** High-frequency linear probe at 2-cm depth showing the fluid collection (indicator facing the patient’s head). An echogenic strip at 0.75-cm depth corresponds to the hilum of the lymph node (white arrow), as well as a small defect through which pus can move (black arrow).
